# Indoleamine-2,3-dioxygenase, an immunosuppressive enzyme that inhibits natural killer cell function, as a useful target for ovarian cancer therapy

**DOI:** 10.3892/ijo.2011.1295

**Published:** 2011-12-13

**Authors:** DONGDONG WANG, YASUSHI SAGA, HIROAKI MIZUKAMI, NAOTO SATO, HIROAKI NONAKA, HIROYUKI FUJIWARA, YUJI TAKEI, SHIZUO MACHIDA, OSAMU TAKIKAWA, KEIYA OZAWA, MITSUAKI SUZUKI

**Affiliations:** 1 Department of Obstetrics and Gynecology, School of Medicine, Jichi Medical University, Tochigi, Japan; 2 Division of Genetic Therapeutics, Center for Molecular Medicine, School of Medicine, Jichi Medical University, Tochigi, Japan; 3 Department of Obstetrics and Gynecology, Shengjing Hospital of China Medical University, Shenyang, P.R. China; 4 National Institute for Longevity Sciences, National Center for Geriatrics and Gerontology, Obu, Japan

**Keywords:** ovarian cancer, indoleamine-2, 3-dioxygenase, peritoneal dissemination, natural killer cell, short hairpin RNA

## Abstract

This study examined the role of the immunosuppressive enzyme indoleamine-2,3-dioxygenase (IDO) in ovarian cancer progression, and the possible application of this enzyme as a target for ovarian cancer therapy. We transfected a short hairpin RNA vector targeting IDO into the human ovarian cancer cell line SKOV-3, that constitutively expresses IDO and established an IDO downregulated cell line (SKOV-3/shIDO) to determine whether inhibition of IDO mediates the progression of ovarian cancer. IDO downregulation suppressed tumor growth and peritoneal dissemination *in vivo*, without influencing cancer cell growth. Moreover, IDO downregulation enhanced the sensitivity of cancer cells to natural killer (NK) cells *in vitro*, and promoted NK cell accumulation in the tumor stroma *in vivo*. These findings indicate that downregulation of IDO controls ovarian cancer progression by activating NK cells, suggesting IDO targeting as a potential therapy for ovarian cancer.

## Introduction

Ovarian cancer is the fifth leading cause of cancer-related-death in the US. Approximately 22,000 women suffered from ovarian cancer in 2010, about 14,000 of whom died of this disease (1). Since most patients with early-stage ovarian cancer seldom have any symptoms, at the time of diagnosis, over 75% are already in advanced stages with peritoneal dissemination and ascites, which are the typical symptoms (2). The standard treatment for ovarian cancer is cytoreductive surgery with platinum/taxane combination chemotherapy. Ovarian cancer is mostly sensitive to chemotherapy (3,4), but becomes ineffective over time due to the development of chemoresistance. The 5-year survival rate is only 40%, and has not improved in the last decade (1). Therefore, new strategies, such as immunotherapy and molecular-targeted therapy, may prove useful in improving the prognosis of ovarian cancer. The most common form of ovarian cancer spread is peritoneal dissemination (2). Although the mechanism involved in this process are largely unknown, studies indicate that, immunotolerance induction plays an important role (5,6).

Indoleamine-2,3-dioxygenase (IDO) is an enzyme that catalyzes the first and rate-limiting step in the kynurenine pathway of tryptophan catabolism. IDO was originally discovered in 1967 (7,8) in the rabbit small intestine and was first purified in 1978 (9). Subsequently, it was reported that IDO could be induced in the mouse lung with either influenza virus infection (10) or bacterial endotoxin shock (11). Proinflammatory mediators, such as interferon-γ or other cytokines can also stimulate IDO induction (12). The first study that described IDO as an immunosuppressant found that IDO in the mouse placenta prevented rejection of the allogeneic fetus (13). Recently, it was clarified that IDO can induce immunotolerance in patients with autoimmune diseases (14–17), chronic infections (18), and cancer (19). It was also reported that most human tumors express IDO (19) and that IDO can contribute to tumor-induced immunosuppression by starving T cells, which are sensitive to tryptophan deficiency. In this situation, tumor cells can escape immune surveillance via the action of IDO (13). Natural killer (NK) cells are important members of the innate immune system, which plays a role in inhibiting the growth and dissemination of several kinds of tumors (20). A series of receptors expressed by NK cells are known to modulate the cytotoxicity of NK cells against tumors (21). The tryptophan-derived catabolic kynurenine can reduce NK cell number and weaken NK cell cytotoxicity by inhibiting NK cell receptors, thus contributing to tumor progression (22). IDO is frequently expressed in many cancers such as gastric, pancreatic, colorectal, and prostate cancers (19). In the gynecological field, IDO expression has been observed in cervical, endometrial, and ovarian cancer (19), and associations between its expression and the prognosis of these cancers have been reported (23–26).

RNA interference (RNAi) is a good technique for gene silencing, that involves a post-transcriptional gene-silencing mechanism (27). Among the different types of RNAi techniques, the use of small interfering RNAs (siRNAs) effectively suppresses gene expression, but the suppression is transient (28), which limits its therapeutic use. Short hairpin RNAs (shRNAs) driven by polymerase III promoters have been developed as an alternative strategy to attain long-term stable target gene silencing and understand the consequence of stable silencing (29,30).

In this study, we used an shRNA vector targeting IDO to silence IDO expression in an IDO-expressing ovarian cancer cell line to clarify the relationship between IDO expression and peritoneal dissemination of ovarian cancer. Moreover, we investigated the function of NK cells in ovarian cancer progression in order to develop an IDO-targeted molecular therapy that inhibits peritoneal dissemination.

## Materials and methods

### Cell culture

The human ovarian cancer cell line SKOV-3 (31) (American Type Culture Collection, Manassas, VA) were cultured in RPMI-1640 medium (Gibco, Grand Island, NY) containing 10% inactivated fetal calf serum (Sigma, St. Louis, MO), 100 U/ml penicillin, and 100 μg/ml streptomycin (Gibco) at 37°C in a 5% CO_2_ atmosphere for no longer than 8 weeks after recovery from frozen stocks.

The NK cell line KHYG-1 (32) was purchased from the Japanese Collection of Research Bioresources (JCRB, Osaka, Japan). Cells were cultured in RPMI-1640 medium supplemented with 100 nM of human interleukin-2 (R&D Systems, Minneapolis, MN) and 10% inactivated fetal calf serum (Sigma), at 37°C in a 5% CO_2_ atmosphere for no longer than 8 weeks after recovery from frozen stocks.

### Antibodies

Anti-human IDO monoclonal antibody was prepared as previously reported (33). Anti-human actin antibody (Sigma) and anti-mouse CD49b antibody (R&D) were used according to the manufacturer’s protocols.

### shRNA stable cell line and control cell line

The DNA oligonucleotides encoding shRNA targeting the IDO gene (forward: 5′-CACCGGGGCAGATTATAAGAATTACGTGTGCTGTCC GTAATTCTTGTAGTCTGCTCCTTTTT-3′, reverse: 5′-CCC CGTCTAATATTCTTAATGCACACGACAGGCATTAAGA ACATCAGACGAGGAAAAATACG-3′) were synthesized, annealed, and inserted into the *Bsp*MI site of the vector piGENE PURhU6 (34), which contained a human U6 promoter, and a puromycin resistance gene. The shRNA expression plasmid (piGENE PURhU6/shIDO) and control plasmid (piGENE PURhU6) were transfected into SKOV-3 using Lipofectamine LTX and Plus Reagent (Invitrogen, Carlsbad, CA) according to the manufacturer’s instructions. The cells were selected using 0.5 μg/ml puromycin (Calbiochem, Darmstadt, Germany). Resistant clones were obtained after 4 weeks as SKOV-3/shIDO, SKOV-3/Mock. The cells were subsequently maintained in the presence of 0.5 μg/ml puromycin.

### Western blotting

Protein (10 μg) extracted from a homogenate of cultured cells was mixed with 2X SDS-PAGE sample buffer [120 mM Tris-HCl (pH 6.8), 4% SDS, 20% glycerol, 0.004% bromophenol blue, and 10% 2-mercaptoethanol]. The mixture was heated at 95°C for 2 min, and electrophoresed on a 0.1% SDS-10% polyacrylamide gel, and then the proteins were blotted onto a polyfluorovinylidene membrane. The menbranes were blocked with Non-Protein Blocking Agent (ATTO Corporation, Tokyo, Japan) at room temperature for 1 h, and incubated with anti-human IDO monoclonal antibody (1:1,000) and anti-human actin polyclonal antibody (1:200) for 1 h at room temperature. The membrane was washed with phosphate-buffered saline (PBS)-Tween-20 three times, and then incubated with horseradish peroxidase-conjugated secondary anti-mouse antibody (Thermo, Rockford, IL) or anti-rabbit antibody (Thermo). Signals were detected by chemiluminescence (ECL kit; Amersham Biosciences, Piscataway, NJ) on X-ray film.

### In vitro cell growth kinetics

SKOV-3/shIDO and SKOV-3/Mock cells (500 of each line) were seeded into a 96-well plate, and cultured in RPMI-1640 medium containing 10% fetal calf serum. Every 24 h, cells were counted using a colorimetric assay with the Cell Proliferation kit II (XTT) (Boehringer Mannheim GmbH Biochemica, Mannheim, Germany), and a growth curve was drawn from the results.

### Sensitivity of transfectants to NK cells in vitro

The sensitivity of SKOV-3/shIDO and SKOV-3/Mock cells to NK cells was investigated by colorimetric assay using XTT. SKOV-3/shIDO and SKOV-3/Mock cells (500 of each line) were seeded into a 96-well plate and co-cultured with KHYG-1 cells (0, 500, 1000, or 2000 cells) in RPMI-1640 medium containing 10% fetal calf serum for 72 h. After 3 washes with PBS to exclude KHYG-1 cells completely, the viable cell count was determined by colorimetric assay and calculated as the percent of control cells (cultured without KHYG-1 cells).

### Experimental animals

Four- to six-week-old female BALB/c nude mice (Japan Clea Laboratories, Tokyo, Japan) were used. All animal experiments were conducted according to the institutional and national guidelines for animal experiments.

### Subcutaneous tumor growth in vivo

SKOV-3/shIDO and SKOV-3/Mock cells (5×10^6^ cells of each line) were inoculated subcutaneously into the back of mice to induce tumor growth. The tumor volume [(long diameter) × (short diameter)^2^ × 1/2] was measured twice a week to draw a tumor growth curve.

### Peritoneal dissemination in vivo

SKOV-3/shIDO and SKOV-3/Mock cells (5×10^6^ cells of each line) were injected intraperitoneally into nude mice, and the mice were observed until death. A survival curve was constructed using the Kaplan-Meier method. The mice were checked for survival twice a day.

### Immunohistochemical staining

At one week after subcutaneous tumor cell inoculation, mice were sacrificed under isoflurane anesthesia, and the tumor was removed. After formalin fixation, paraffin sections were prepared, deparaffinized, and treated with hydrogen peroxide for 30 min to block endogenous peroxidase. The sections were then reacted with a 1:10 dilution (5 μg/ml) of anti-mouse CD49b primary antibody for 16 h at room temperature, washed 3 times washes with PBS, and then incubated with enzyme-conjugated streptavidin for 30 min. The sections were again washed with PBS 3 times, and color was developed using the diaminobenzidin method. The number of stained NK cells was counted under high-power magnification (x400).

### Statistical analysis

Except for the comparison of survival curves, the test of significance between the 2 groups was performed using Student’s t-test. The generalized Wilcoxon test was used to compare survival curves between the 2 groups. A P-value of <0.05 was considered significant.

## Results

### Establishing an IDO-downregulated cell line

**Figure 1:**
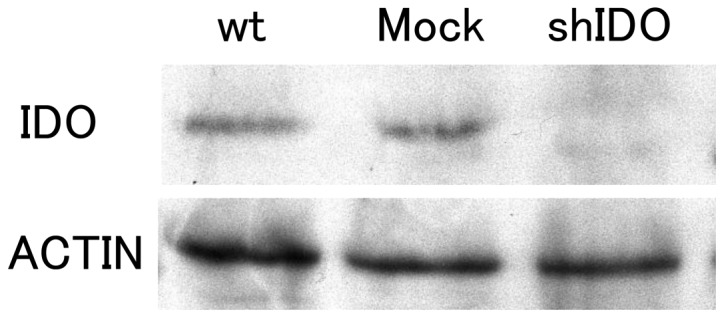
Western blot of parental cells (wt) and control vector-transfected cells (Mock) showing evident IDO expression. In contrast, the shIDO vector-transfected cells (shIDO) did not show IDO expression.

Fig. 1 shows the results of Western blot analysis of the shIDO expression vector- or control vector-transfected ovarian cancer cell line SKOV-3. Parental cells (wt) and control vector-transfected cells (Mock) showed evident IDO expression. In contrast, the shIDO expression vector-transfected cells (shIDO) did not show IDO expression, confirming IDO downregulation in the SKOV-3/shIDO cell line.

### In vitro cell growth kinetics

**Figure 2:**
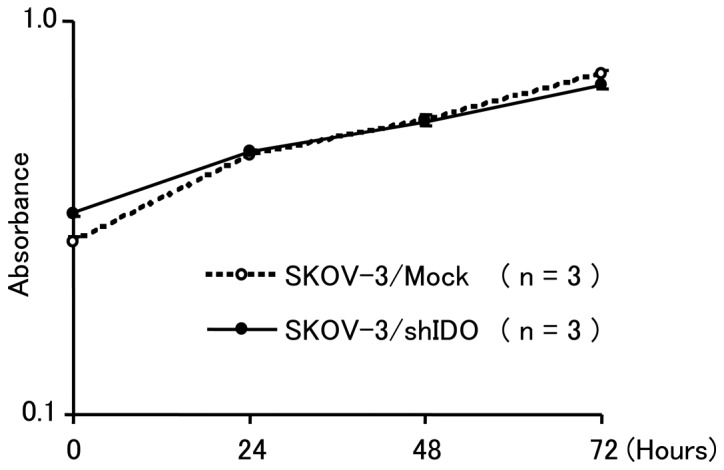
Cell growth curves of SKOV-3/shIDO and SKOV-3/Mock (control) cells. There was no significant difference between the 2 groups. Results are expressed as mean ± SD.

Growth curve analyses of SKOV-3/shIDO and SKOV-3/Mock cells showed no significant difference between the two groups, suggestiong that the downregulation of IDO did not affect cell growth *in vitro* (Fig. 2).

### Sensitivity of transfectants against NK cells in vitro

**Figure 3:**
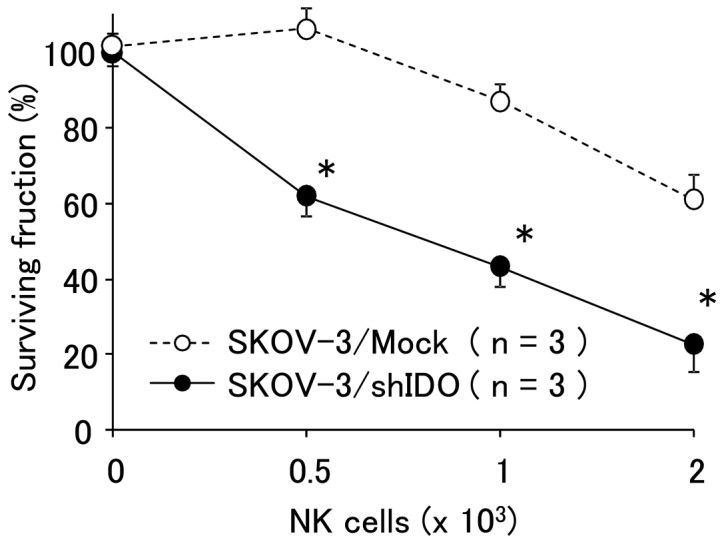
The percent of viable tumor cells co-cultured with NK cells. The percent survival of SKOV-3/shIDO cells was significantly lower than that of control cells. ^*^P<0.01. The results are expressed as mean ± SD.

The proportion of viable tumor cells co-cultured with NK cells is shown in Fig. 3. The percent survival of SKOV-3/shIDO cells was significantly lower than that of the control cells, indicating that the downregulation of IDO reinforced the sensitivity of tumor cells against NK cells.

### Tumor growth in vivo

**Figure 4:**
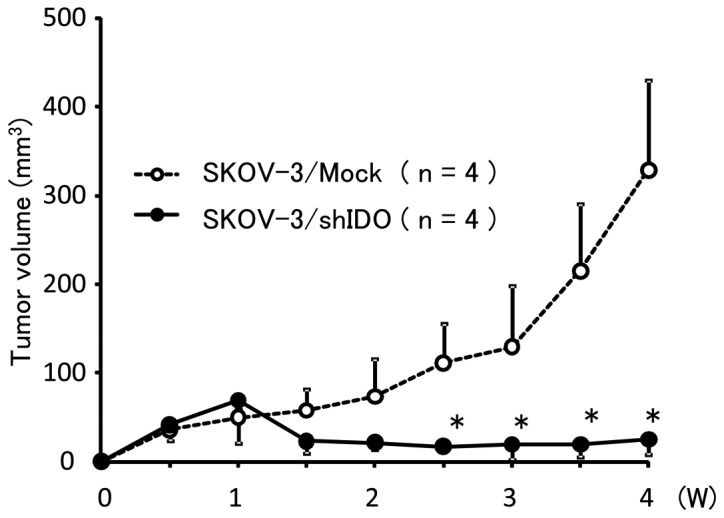
Subcutaneous tumor growth curves of SKOV-3/shIDO and control cells. Both groups of cells formed small nodules one week after inoculation. Subsequently, the tumors in the control group enlarged, whereas those in the SKOV-3/shIDO group disappeared. ^*^P<0.05. Mean ± SD.

Both SKOV-3/shIDO and control cells formed small nodules one week after inoculation (Fig. 4). Subsequently, the tumors in the control group were enlarged, whereas those in the SKOV-3/shIDO group were reduced, suggesting that the downregulation of IDO inhibited tumor growth *in vivo*.

### Number of NK cells in the tumor stroma

**Figure 5:**
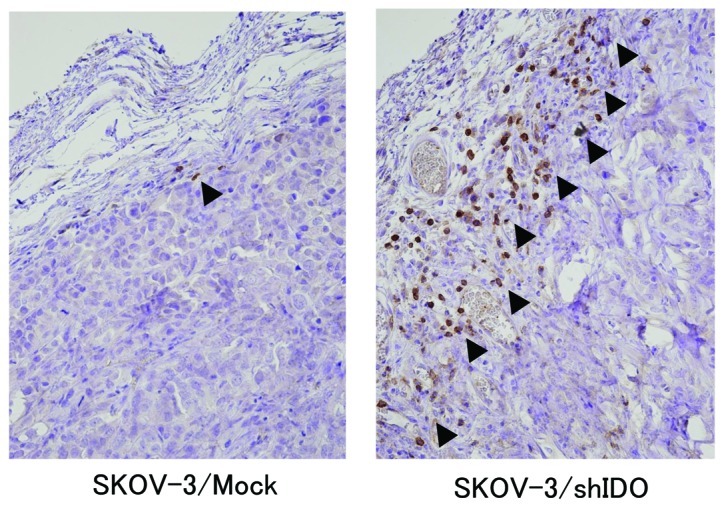
CD49b expression in SKOV-3/shIDO and control subcutaneous tumors. The black arrowheads indicate NK cells accumulating in the tumor stroma.

**Figure 6:**
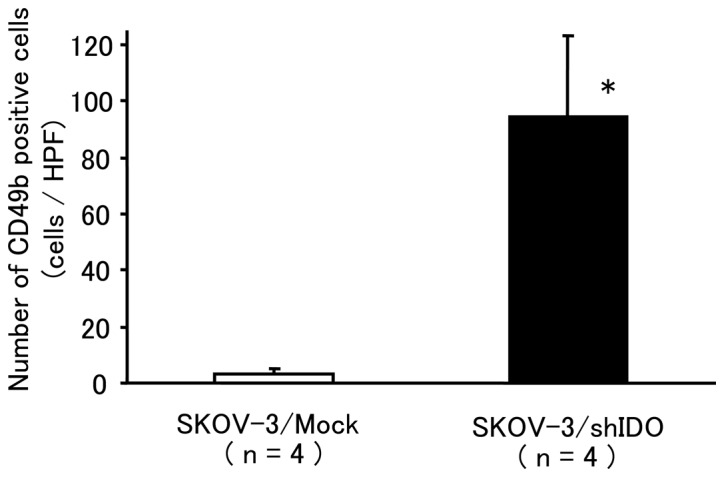
The number of NK cells per high-power field. The number of NK cells (94±29) that accumulated in the SKOV-3/shIDO tumors was significantly higher than that (3±2) in the control tumors. ^*^P<0.01. Mean ± SD.

Immunostaining of NK cells (black arrowhead) shows accumulation of NK cells in the stroma of SKOV-3/shIDO and control subcutaneous tumors (Fig. 5). The number of NK cells (94±29) that accumulated in the SKOV-3/shIDO tumors was significantly higher than that (3±2) in the control tumors (P<0.01) (Fig. 6). These results suggest that the downregulation of IDO promoted NK cell accumulation around the tumor.

### Peritoneal dissemination in vivo

**Figure 7:**
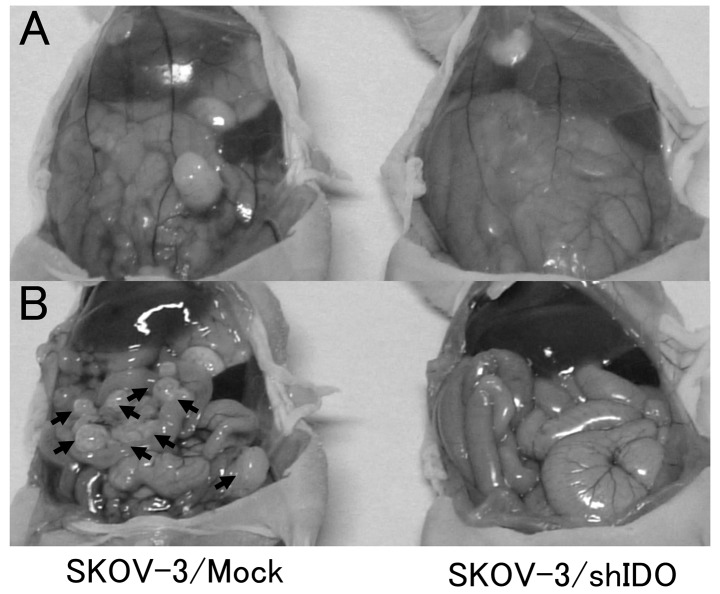
Peritoneal dissemination and ascites accumulation at 4 weeks after the intraperitoneal inoculation of SKOV-3/shIDO or control cells. Ascites accumulation (A). Peritoneal dissemination (B). The black arrow indicates disseminated peritoneal tumors.

**Figure 8:**
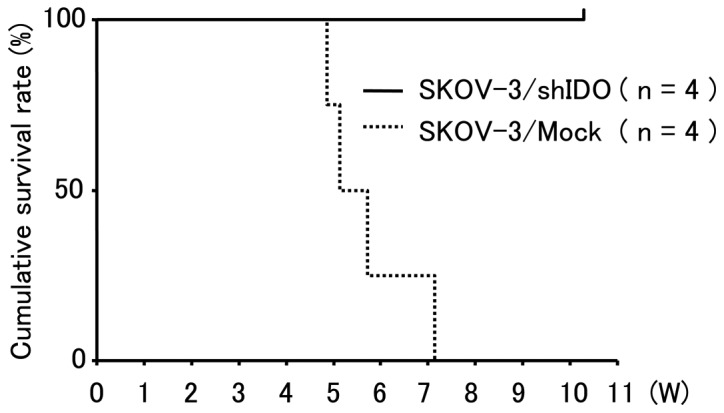
Survival curves of intraperitoneally inoculated mice. All control cell-inoculated mice died of peritoneal dissemination with ascites within 50 days after inoculation, whereas all SKOV-3/shIDO cell-inoculated mice survived for longer than 70 days. ^*^P<0.01.

Four weeks after intraperitoneal tumor cell inoculation, mice with intraperitoneally-injected control cells demonstrated bloody ascites and marked peritoneal dissemination, whereas those receiving the intraperitoneal injection of SKOV-3/shIDO cells showed no abnormal changes (Fig. 7A and B). All control cell-inoculated mice died of peritoneal dissemination with ascites within 50 days after inoculation, whereas all SKOV-3/shIDO cell-inoculated mice survived longer than 70 days after inoculation (P<0.01) (Fig. 8). Thus, downregulating IDO inhibited peritoneal dissemination formation and ascites accumulation in tumor-inoculated mice.

## Discussion

The experiments described herein aimed to clarify the relationship between the immunosuppressive enzyme IDO and ovarian cancer progression, as well as to develop a molecular therapy-targeting IDO. Previously, we transfected an IDO expression vector into a non-IDO-expressing human ovarian cancer cell line and established an IDO-expressing cell line to examine the relationship between IDO expression and ovarian cancer progression, especially in term of peritoneal dissemination *in vivo* (35). In the present study, we utilized an shRNA expression vector targeting the IDO gene to examine whether inhibition of IDO can control peritoneal dissemination of ovarian cancer. We found that the downregulation of IDO expression did not influence cancer cell growth *in vitro*, but controlled tumor growth and peritoneal dissemination *in vivo*. In addition, the downregulation of IDO reinforced the sensitivity of cancer cells to NK cells *in vitro* and promoted NK cell accumulation in the tumor stroma *in vivo*. These findings indicate that the downregulation of IDO controls peritoneal dissemination of ovarian cancer by promoting NK cell accumulation in tumors, suggesting that IDO is a useful therapeutic target for patients with ovarian cancer.

Lack of the essential amino acid tryptophan and accumulation of its metabolite, kynurenine, inhibit cell growth and induce apoptosis. T cells are particularly sensitive to this type of stress (13). Regarding the mechanism of cancer cell immunotolerance, IDO has been shown to promote local tryptophan depletion, resulting in T-cell function suppression around IDO-expressing cancer cells and local immunotolerance (19). The possibility cannot be excluded that IDO expression is involved in the immunotolerance of ovarian cancer through such a T cell-mediated mechanism. We initially obtained a murine ovarian tumor cell line (OV2944-HM-1) with the ability to develop into subcutaneous tumor and disseminate peritoneally in immunocompetent mice. However, IDO was hardly detected in this cell line, according to the results of Western blot analysis using an anti-mouse IDO antibody (data not shown). Therefore, we chose the human ovarian cancer cell line (SKOV-3) that constitutively expresses IDO and implanted them in nude mice. Nude mice congenitally lack T cells; therefore, in this experimental system, we could not examine the effect of IDO on T-cell function.

It has been reported that IDO induces the accumulation of the tryptophan metabolite kynurenine, which suppresses NK cell receptor expression, and thereby inhibits NK cell function (22). Similarly, in our previous experiments, IDO expression inhibited the cytotoxic activity of NK cells *in vitro* and suppressed NK cell accumulation in the tumor stroma *in vivo* (35). Herein, we demonstrated that IDO downregulation enhanced the sensitivity of cancer cells to NK cells *in vitro* and promoted NK cell accumulation in the tumor stroma *in vivo*. Thus, the downregulation of IDO reinforced the sensitivity of cancer cells to NK cells, mediating peritoneal dissemination and growth of ovarian cancer.

Typical methods of inhibiting IDO function include the use of 1-methly-tryptophan (1-MT) and gene silencing by RNAi. In IDO-catalyzed tryptophan metabolism, 1-MT competes with tryptophan for IDO, acting as an IDO inhibitor (36). Inaba *et al* reported that the oral administration of 1-MT to the host suppressed the tumor growth of IDO-overexpressing ovarian cancer cells with enhanced proliferative activity (26). Similarly, in our previous study, we showed that oral administration of 1-MT inhibited the tumor growth potential of IDO-transfected ovarian cancer cells with enhanced proliferative activity (35). In our study, mice given 1-MT orally showed no fatal side effects (35). These findings suggest the possibility of IDO-targeted molecular therapy for ovarian cancer using the oral administration of 1-MT or its analogues. Muller *et al* reported that the combination of 1-MT with paclitaxel synergistically regressed an autochthonous breast cancer (37). In addition, Inaba *et al* demonstrated that treatment with 1-MT plus paclitaxel synergistically prolonged mouse survival compared to treatment with paclitaxel alone in an IDO-overexpressing ovarian cancer peritoneal carcinomatosis model (26). Since paclitaxel is a key drug in the chemotherapy of ovarian cancer, the combined use of such an anticancer drug and targeted therapy against IDO may be advantageous in treating ovarian cancer.

Compared to 1-MT treatment, RNAi demonstrates higher potency and efficiency (38). To date, chemically synthesized siRNA and vector-mediated expression of shRNA are the more commonly used RNAi techniques for gene silencing in mammalian cells (30,39). Although siRNA can be more easily transfected into cancer cells, and its silencing function is more effective, its function is transient. The remarkable advantages of shRNA is that the inhibition of target genes can last for weeks or even months, making it possible to elucidate the consequences of long-term stable silencing of a gene (30). In actual clinical settings, nanoparticle-based vectors (40) or viral-based expression vectors could be used to deliver the IDO shRNA to the cancer cells.

The results of this study demonstrate that the downregulation of IDO in human ovarian cancer cells constitutively expressing IDO inhibits ovarian cancer progression, suggesting that the use of IDO-targeted shRNA as a potentially effective molecular-targeted therapy for ovarian cancer.
